# Metabolic disease and ABHD6 alter the circulating bis(monoacylglycerol)phosphate profile in mice and humans[S]

**DOI:** 10.1194/jlr.M093351

**Published:** 2019-03-20

**Authors:** Gernot F. Grabner, Nermeen Fawzy, Maria A. Pribasnig, Markus Trieb, Ulrike Taschler, Michael Holzer, Martina Schweiger, Heimo Wolinski, Dagmar Kolb, Angela Horvath, Rolf Breinbauer, Thomas Rülicke, Roland Rabl, Achim Lass, Vanessa Stadlbauer, Birgit Hutter-Paier, Rudolf E. Stauber, Peter Fickert, Rudolf Zechner, Gunther Marsche, Thomas O. Eichmann, Robert Zimmermann

**Affiliations:** Institute of Molecular Biosciences,* University of Graz, Graz, Austria; Division of Pharmacology† Otto Loewi Research Center, Medical University of Graz, Graz, Austria; Core Facility Ultrastructure Analysis§ Division of Cell Biology, Histology and Embryology, Gottfried Schatz Research Center, Medical University of Graz, Graz, Austria; Division of Gastroenterology and Hepatology,** Department of Internal Medicine, Medical University of Graz, Graz, Austria; Institute of Organic Chemistry†† Graz University of Technology, Graz, Austria; Institute of Laboratory Animal Science§§ University of Veterinary Medicine Vienna, Vienna, Austria; QPS Austria GmbH,*** Grambach, Austria; Center for Explorative Lipidomics††† Graz, Austria; BioTechMed-Graz§§§ Graz, Austria

**Keywords:** nonalcoholic fatty liver disease, obesity, lysosomal storage disorders, phospholipids, lysobisphosphatidic acid, α/β-hydrolase domain-containing 6, lipase

## Abstract

Bis(monoacylglycerol)phosphate (BMP) is a phospholipid that is crucial for lipid degradation and sorting in acidic organelles. Genetic and drug-induced lysosomal storage disorders (LSDs) are associated with increased BMP concentrations in tissues and in the circulation. Data on BMP in disorders other than LSDs, however, are scarce, and key enzymes regulating BMP metabolism remain elusive. Here, we demonstrate that common metabolic disorders and the intracellular BMP hydrolase α/β-hydrolase domain-containing 6 (ABHD6) affect BMP metabolism in mice and humans. In mice, dietary lipid overload strongly affects BMP concentration and FA composition in the liver and plasma, similar to what has been observed in LSDs. Notably, distinct changes in the BMP FA profile enable a clear distinction between lipid overload and drug-induced LSDs. Global deletion of ABHD6 increases circulating BMP concentrations but does not cause LSDs. In humans, nonalcoholic fatty liver disease and liver cirrhosis affect the serum BMP FA composition and concentration. Furthermore, we identified a patient with a loss-of-function mutation in the *ABHD6* gene, leading to an altered circulating BMP profile. In conclusion, our results suggest that common metabolic diseases and ABHD6 affect BMP metabolism in mice and humans.

Bis(monoacylglycerol)phosphate (BMP), also known as lysobisphosphatidic acid, is a phospholipid consisting of two phosphate-linked glycerol molecules in an unusual *sn*-1 glycerophospho-s*n*-1′ glycerol stereoconformation, with each glycerol esterified with a single FA (1). BMP is found in the intraluminal vesicles (ILVs) of late endosomes and lysosomes and plays an important role in their structure and function. It is produced during the maturation of endosomes, which acquire ILVs through the inward budding of the outer membrane, leading to a multivesicular appearance. BMP favors the formation of ILVs (2) and accounts for approximately 15 mol% of total phospholipids in late endosomes (3). Due to its exceptional structure, BMP is resistant to degradation by acid phospholipases and forms a docking station for lysosomal proteins. It is negatively charged at an acidic pH, facilitating the binding of positively charged hydrolases and lipid-binding proteins to ILVs (4). Based on these properties, BMP plays a central role in cargo sorting by stimulating the degradation and sorting of lipids.

Various genetic lysosomal storage disorders (LSDs), such as Niemann-Pick disease, neuronal ceroid lipofuscinoses, and Wolman disease, are associated with BMP accumulation in tissues (5). These diseases are characterized by the accumulation of undigested material in lysosomes due to a deficiency of degradative enzymes or defects in lysosomal transport. Tissue BMP accumulation occurs secondary to a “traffic jam” in acidic organelles and counteracts the pathological accumulation of lipids in these organelles (6, 7). In LSDs, BMP is also elevated in the circulation and can be used as a diagnostic biomarker (8, 9). Analyzing serum BMP concentrations is particularly important in the diagnosis of a drug-induced LSD, also designated as drug-induced phospholipidosis. This disorder is caused by cationic amphiphilic drugs (CADs) that accumulate within and disturb the function of lysosomes (10). Today, almost 50 clinically relevant drugs are considered as CADs, and drug-induced phospholipidosis has become a major concern in drug development (11, 12).

Despite its important role in physiology and pathophysiology, major aspects of BMP metabolism in health and disease remain elusive. Genes involved in BMP synthesis and degradation are largely unknown. We previously demonstrated that the serine hydrolase α/β-hydrolase domain-containing 6 (ABHD6) exhibits BMP hydrolase activity in vitro and in vivo (13). Antisense oligonucleotide-mediated knockdown of ABHD6 in mice resulted in elevated hepatic BMP content, which was further increased upon high-fat diet (HFD) feeding (13). ABHD6 was originally described as a monoglyceride (MG) hydrolase with an important function in endocannabinoid signaling (14). More recent studies, however, have shown its involvement in intracellular vesicle transport (15, 16), energy and lipid metabolism (17, 18), and tumor metastasis (19). Although several loss-of-function SNPs are known for human ABHD6 (13), so far no patient with ABHD6 deficiency has been described.

In this study, we investigated whether lipid-enriched diets, frequently used in obesity and atherosclerosis research, affect BMP metabolism in mice. Furthermore, we analyzed the circulating BMP profile of patients with liver disease and characterized the role of ABHD6 in BMP metabolism in mice and humans.

## MATERIALS AND METHODS

### Animals

Animal experiments were approved by the Austrian Federal Ministry for Science, Research, and Economy (protocol numbers BMWF-66.007/7-ll/3b/2013 and BMWF-66.007/0002-ll/3b/2014) and the ethics committee of the University of Graz and conducted in compliance with the Council of Europe Convention (ETS 123). All mice were bred and maintained on a regular dark-light cycle (14 h dark, 10 h light) at 22 ± 1°C in a barrier facility in specific pathogen-free quality. A standard laboratory chow diet (Ssniff Spezialdiäten GmbH, Soest, Germany) and drinking water were provided ad libitum unless otherwise indicated. ABHD6-deficient mice were generated using embryonic stem cells from EUCOMM (*Abhd6^tm1a^*^(EUCOMM)Hmgu^) and crossbred with transgenic animals expressing Cre recombinase under the control of the cytomegalovirus promotor, resulting in heterozygous ABHD6-deficient mice. Heterozygous mutants were bred to obtain homozygous ABHD6 KO mice and WT control littermates. For all studies, age-matched male mice were used.

### Dietary intervention

The standard chow diet was supplemented with amiodarone to obtain a dietary intake of 100 mg amiodarone/kg/day per mouse, and the mice were fed for 3 consecutive days. For dietary intervention, 6-week-old mice were fed an HFD or Western-type diet (WTD) (Ssniff Spezialdiäten GmbH) for 12 weeks.

### Metabolic phenotyping

To determine the food intake, locomotor activity, oxygen consumption, and respiratory quotient of mice, the LabMaster phenotyping platform (TSE Systems, Bad Homburg, Germany) was used. Therefore, 3-month-old mice were accustomed to LabMaster drinking bottles for at least 3 days in their home cages. Mice were then housed singly and acclimatized to phenotyping cages for 3 days before measurement.

### Cloning and protein expression

The cloning of β-galactosidase (LacZ) and human ABHD6 (NP_065727) has previously been described (13). Single-nucleotide exchange in human ABHD6 was performed with the Q5 Site-Directed Mutagenesis Kit (New England Biolabs, Ipswich, MA) using the following primers: 5′-CAGTTCTGTTCTTCCTTCCGG-3′ (forward) and 5′-ATAGTCTTCATGGTGAAC-3′ (reverse). COS-7 cells (SV-40 transformed monkey embryonic kidney cells; ATCC, Manassas, VA) were cultivated in DMEM (Invitrogen, Carlsbad, CA) containing 10% FCS (Sigma-Aldrich, St. Louis, MO) under standard conditions (95% humidified atmosphere, 37°C, 5% CO_2_). Cells were transfected with recombinant DNA complexed to Metafectene (Biontex Laboratories GmbH, München, Germany) in FCS-free medium. After 4 h, the medium was changed to serum-containing medium, and cells were harvested 48 h posttransfection.

### Immunoblotting

Tissues were homogenized in RIPA buffer, and cells were lysed in extraction buffer [0.25 M sucrose, 1 mM EDTA, 1 mM Tris/HCl (pH 8), 50 mM NaCl, 0.1% Nonidet P-40, 20 µg/ml leupeptin, 2 µg/ml antipain, and 1 µg/ml pepstatin]. Ten to thirty micrograms of protein were subjected to SDS-PAGE, transferred to a PVDF membrane (Karl Roth GmbH, Karsruhe, Germany), and blocked with 10% blotting-grade milk powder (Karl Roth GmbH) in TST (50 mM Tris/HCl, 0.15 M NaCl, 0.1% Tween-20, pH 7.4). Membranes were incubated with antibodies against ABHD6 (17), GAPDH (2118S; Cell Signaling Technology, Danvers, MA), β-2-glycoprotein I (β2GPI) (20), CD63 (21), or His-Tag (18184; Abcam, Cambridge, UK) prepared in 5% milk powder in TST. Antibody binding was detected with anti-rabbit (7074; Cell Signaling Technology) or anti-mouse (NA931V; GE Healthcare, Little Chalfont, UK) HRP-linked secondary antibodies in 5% milk powder in TST and visualized using Clarity Western ECL Substrate and the ChemiDoc Touch Imaging System (Bio-Rad, Hercules, CA).

### Targeted BMP analysis

Total lipids of weighed tissue explants were extracted twice according to Folch, Lees, and Sloane Stanley (22) using 4 ml chloroform-methanol (2/1; v/v) containing 500 pmol butylated hydroxytoluene, 1% acetic acid, and 150 pmol 14:0-14:0 BMP as internal standards (Avanti Polar Lipids, Alabaster, AL) per sample. Extraction was performed under constant shaking for 90 min at room temperature (RT). After the addition of 800 µl distilled H_2_O and further incubation for 30 min at RT, samples were centrifuged at 1,000 *g* for 15 min at RT to establish phase separation. The lower organic phase was collected, 2.5 ml chloroform were added to the remaining aqueous phase, and the second extraction was performed as described above (30 min at RT with subsequent centrifugation). Combined organic phases of the double extraction were dried under a stream of nitrogen and resolved in 200 µl methanol/2-propanol/water (6/3/1; v/v/v) for ultra-performance LC/MS analysis.

Chromatographic separation was modified after Knittelfelder et al. (23) using an AQUITY UPLC system (Waters Corporation, Milford, MA) equipped with a Kinetex EVO-C18 column (2.1 × 50 mm, 1.7 µm; Phenomenex, Torrance, CA) starting a 15 min linear gradient with 100% solvent A (methanol-water; 1/1; v/v; 10 mM ammonium acetate, 0.1% formic acid, 8 µM phosphoric acid). An EVOQ Elite triple quadrupole mass spectrometer (Bruker, Billerica, MA) equipped with an ESI source was used for detection. BMP species were analyzed by selected reaction monitoring using [M + NH_4_]^+^ to [RCOO + 58]^+^ (of the respective esterified FA) as a transition (23 eV collision energy, 150 ms cycle time, 0.7 resolution for Q1/Q3). Data were normalized for recovery, extraction, and ionization efficacy by calculating analyte-internal standard ratios, quantified via external calibration using BMP 36:2 (857135; Avanti Polar Lipids), and expressed as mol/g tissue or mol/ml plasma.

### Determination of tissue acylglycerol content

Total lipids were extracted using the method of Folch et al. (22). After centrifugation, the organic phase was collected, dried under a stream of nitrogen, and dissolved in 2% Triton X-100 by sonication. Acylglycerol levels were determined using TG Infinity reagent (Thermo Fisher Scientific, Waltham, MA) and glycerol as a standard.

### FPLC plasma fractionation

Plasma samples were subjected to gel filtration using a Superdex 200 Increase 10/300 GL column in an ÄKTA advanced fast-protein LC (FPLC) system (GE Healthcare) with 0.1 M PBS, pH 7.4, containing 1 mM EDTA. Fractions (800 µl) were collected for further analysis.

### Human serum samples

Blood was taken from patients with liver disease and healthy volunteers in serum tubes (Greiner, Kremsmünster, Austria) after obtaining written informed consent from each patient in agreement with the ethics committee of the Medical University of Graz in accordance with the principles of the Declaration of Helsinki. Blood was sampled from 83 patients with liver disease with clinical and radiological evidence and/or biopsy-proven nonalcoholic fatty liver (NAFL), nonalcoholic steatohepatitis (NASH), and cirrhosis. Patients with a Child-Pugh score >11, abstinence from alcohol for <2 weeks, clinical evidence of active infection, antibiotic treatment within 7 days prior to enrolment (except for primary or secondary prophylaxis of spontaneous bacterial peritonitis), gastrointestinal hemorrhage within the previous 2 weeks, use of immune-modulating agents within 1 month (steroids, etc.), renal failure (such as hepatorenal syndrome), creatinine >1.5× the upper limit of normal, hepatic encephalopathy II to IV, pancreatitis, other organ failure, hepatic or extrahepatic malignancy, and pregnancy were excluded.

Furthermore, blood was sampled from 32 age-matched healthy controls after they passed the following exclusion criteria: any history of cardiovascular disease, pregnancy, obesity, dyslipidemia, liver disease, renal disease, or diabetes or clinical signs of inflammation. Control subjects were free of lipid-lowering medication and anti-inflammatory drugs. Cytokines were quantified using a multiplex bead-based immunoassay (eBioscience, San Diego, CA).

### Statistical analysis

Figures were prepared using GraphPad Prism 6 (GraphPad Software, San Diego, CA). Data sets are represented as means + SDs. Statistical significance between two groups was determined by Student’s unpaired *t*-test (two-tailed) or ANOVA followed by Dunnett’s post hoc test for multiple comparisons.

## RESULTS

### Dietary lipid overload and amiodarone affect hepatic and circulating BMP profiles

**Fig. 1.**
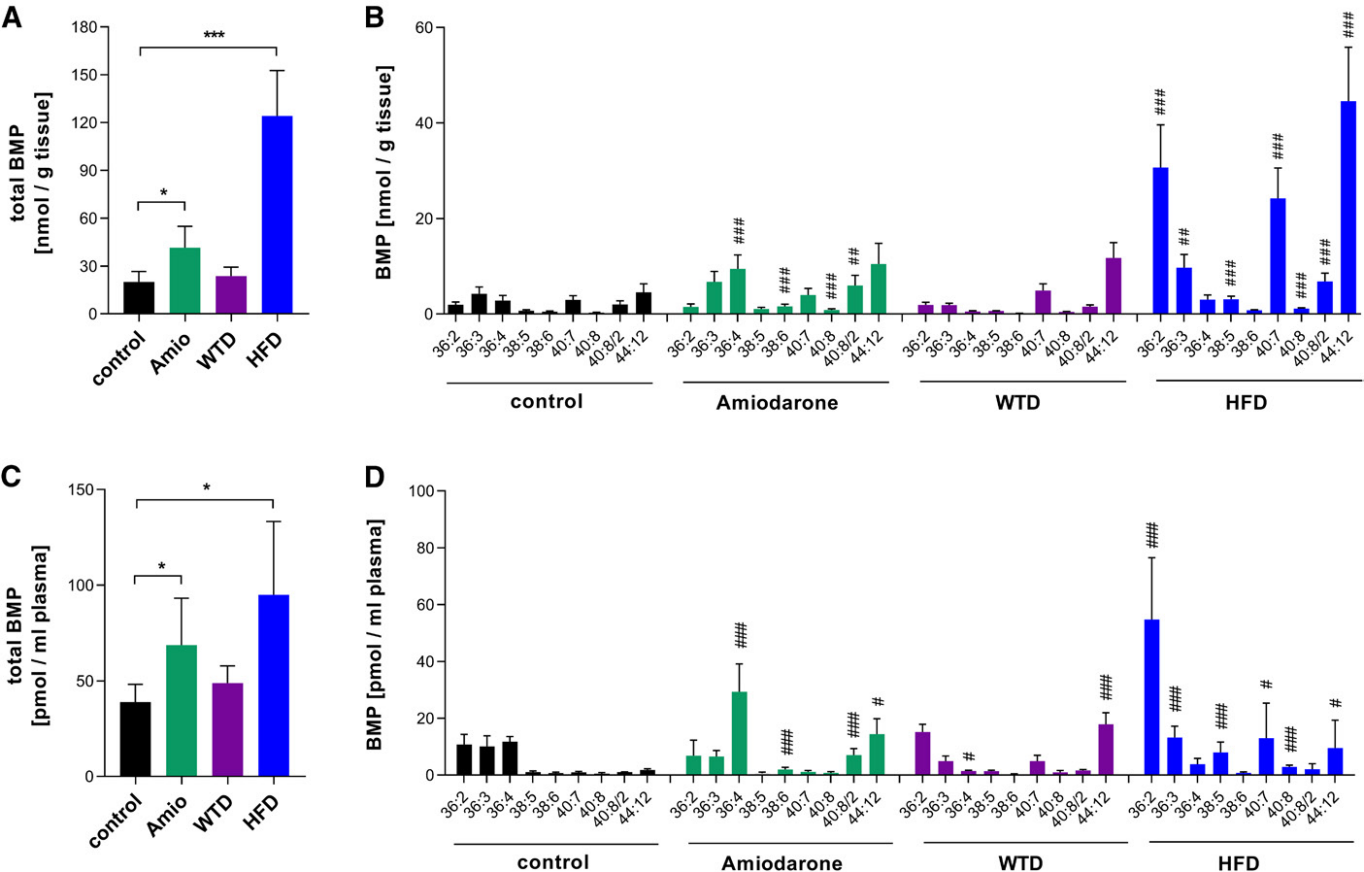
Effects of amiodarone and lipid-rich diets on hepatic and plasma BMP content. Total liver BMP content of mice fed a chow diet (control), 100 mg/kg/day amiodarone for 3 days, a WTD for 3 months, or an HFD for 3 months (A) and respective BMP subspecies (*n* = 4–6) (B). Lipid species are annotated as summarized carbon atoms:double bonds of the attached acyl chains. Total plasma BMP content of mice fed a chow diet (control), amiodarone, WTD, or HFD (C) and respective BMP subspecies (*n* = 5–6) (D). Data are presented as means + SDs. Statistical significance was evaluated by unpaired two-tailed Student’s *t*-test (*P < 0.05, **P < 0.01, and ***P < 0.001 vs. chow-fed controls) or ANOVA followed by Dunnett’s post hoc test (#*P* < 0.05, ##*P* < 0.01, and ###*P* < 0.001 vs. chow-fed controls).

Previous studies demonstrated that CADs affect BMP concentrations in tissues and in the circulation (12, 24). To compare the effects of CADs and widely used experimental lipid-enriched diets on BMP metabolism, we fed mice an HFD or WTD or treated them with the CAD amiodarone. We then determined the concentration and FA composition of BMP in the liver and plasma. Total liver BMP content increased 2- and 6-fold in amiodarone-treated and HFD-fed mice, respectively, while no significant changes were observed in mice fed the WTD (**Fig. 1A**). Notably, different treatments led to distinct alterations in BMP FA composition. In the livers of control mice, the most abundant FAs esterified to BMP were linoleic acid (mostly found in BMP 36:3 and 36:4), DHA (found in BMP 40:7, 40:8/2, and 44:12), and oleic acid (found in BMP 36:2 and 36:3). Amiodarone treatment led to a significant increase in linoleic and arachidonic acid containing BMP subspecies in the liver (BMP 36:4, 38:6, and 40:8), while WTD did not lead to significant alterations. Mice fed an HFD exhibited a substantial increase of most subspecies, with the highest abundance of BMP 36:2, 40:7, and 44:12 (Fig. 1B).

These treatments also led to distinct alterations in the plasma BMP profile. Total plasma BMP concentrations were moderately affected by amiodarone and the WTD and increased 2.5-fold in mice fed the HFD (Fig. 1C). Amiodarone caused an elevation of BMP 36:4, 40:8/2, and 44:12. As observed for amiodarone, the WTD also strongly increased BMP 44:12, while BMP 36:4 was decreased by 80%. The HFD caused an increase of most detected BMP species, with BMP 36:2 being the most abundant species (Fig. 1D). Our results demonstrate that amiodarone and a WTD and HFD cause distinct alterations in hepatic and/or plasma BMP profiles, allowing a clear distinction between different treatments. Circulating BMP profiles under basal and pathophysiological conditions clearly differ from the hepatic profiles. This may be due to both selective release of distinct subspecies and secretion of BMP from the cells of different organs.

### Generation and characterization of global ABHD6-deficient mice

**Fig. 2.**
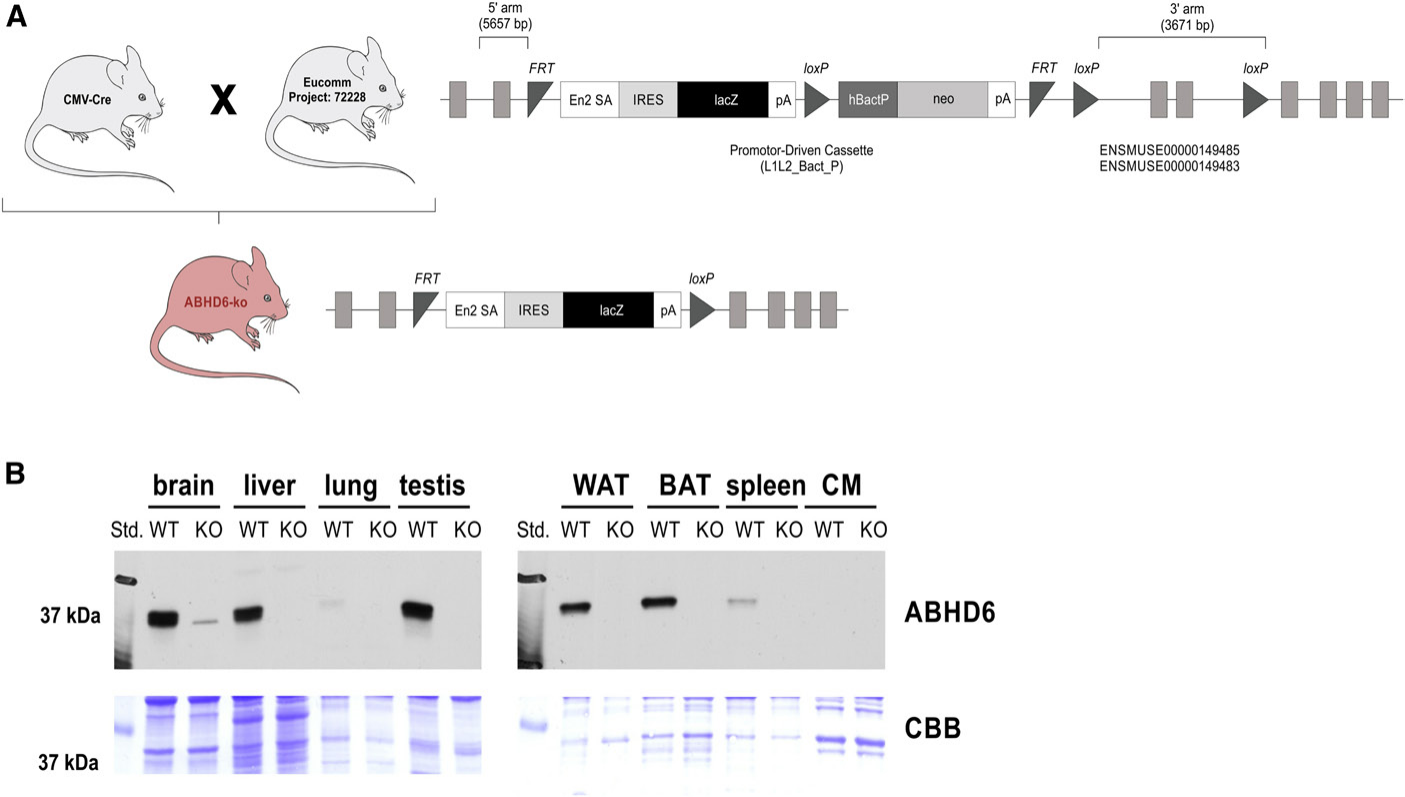
Characterization of mice with global genetic ABHD6 deletion. A: Strategy for generating ABHD6 KO mice. B: Immunoblotting of ABHD6 in tissue lysates of WT and ABHD6 KO mice with CBB staining as a loading control. BAT, brown adipose tissue; CBB, Coomassie Brilliant Blue; WAT, white adipose tissue.

Although changes in BMP concentrations in plasma and tissues are closely linked to lysosomal stress, very little is known about the molecular pathways regulating BMP metabolism. In fact, the rate-limiting enzymes catalyzing BMP synthesis and degradation remain elusive, and the mechanism of BMP secretion has not been investigated. We have previously shown that ABHD6 acts as an intracellular BMP hydrolase affecting hepatic BMP degradation (13). To further investigate the role of ABHD6 in BMP metabolism under physiological and pathophysiological conditions, we generated mice globally lacking ABHD6. Mice were generated using embryonic stem cells from EUCOMM containing a LacZ cassette and a promotor-driven selection cassette (neo) in introns 3 and 4 of the *Abhd6* gene. The selection cassette as well as exons 4 and 5 of *Abhd6* were flanked by loxP sites. Mice bearing the targeted allele were crossed with transgenic mice expressing Cre recombinase under the control of a cytomegalovirus promotor, resulting in the deletion of neo, exon 4, and exon 5 (**Fig. 2A**). The loss of the ABHD6 protein was confirmed by immunoblotting (Fig. 2B). A gross metabolic characterization of ABHD6 KO mice revealed no alterations in food intake, locomotor activity, oxygen consumption, CO_2_ production, and respiratory coefficient compared with WT controls in mice fed the chow diet (supplemental Fig. S1A–E). In accordance with published data, however, we observed that ABHD6 KO mice fed the HFD exhibited reduced body weight and liver acylglycerol content compared with WT controls (supplemental Fig. S1F, G) (17, 18). Resistance against diet-induced obesity has been shown to be caused by increased energy expenditure due to white adipose tissue browning (18). Upon HFD feeding, ABHD6-deficient mice also exhibit reduced de novo lipogenesis in the liver that may counteract hepatic steatosis (17). In contrast to the HFD, no differences between genotypes were observed in the body weight and liver acylglycerol content in mice fed the chow diet or WTD or mice treated with amiodarone (supplemental Fig. S1F, G). ABHD6 has originally been identified as an MG hydrolase (25), with high expression in several tissues, including the brain (17). ABHD6 may thus affect the degradation of the major endocannabinoid 2-archidonoylglycerol (2-AG), which may lead to altered endocannabinoid signaling and neuronal transmission. Analysis of the MG profile in the brain, liver, and plasma revealed unchanged MG concentration and FA composition (supplemental Fig. S2A–C). To exclude that the lack of ABHD6 causes behavioral alterations in mice, we performed several behavioral tests. We assessed anxiety- and depression-like behavior in the elevated plus maze and forced swim test, social interaction in the three-chamber test, and spatial learning and memory performance in the Morris water maze. However, we could not observe differences between WT and ABHD6 KO mice in any of the aforementioned tests (supplemental Fig. S2D–G). These observations indicate that ABHD6 deficiency does not cause severe behavioral or metabolic alterations in mice fed a chow diet.

### ABHD6-deficient mice exhibit unchanged hepatic but increased circulating BMP concentrations

**Fig. 3.**
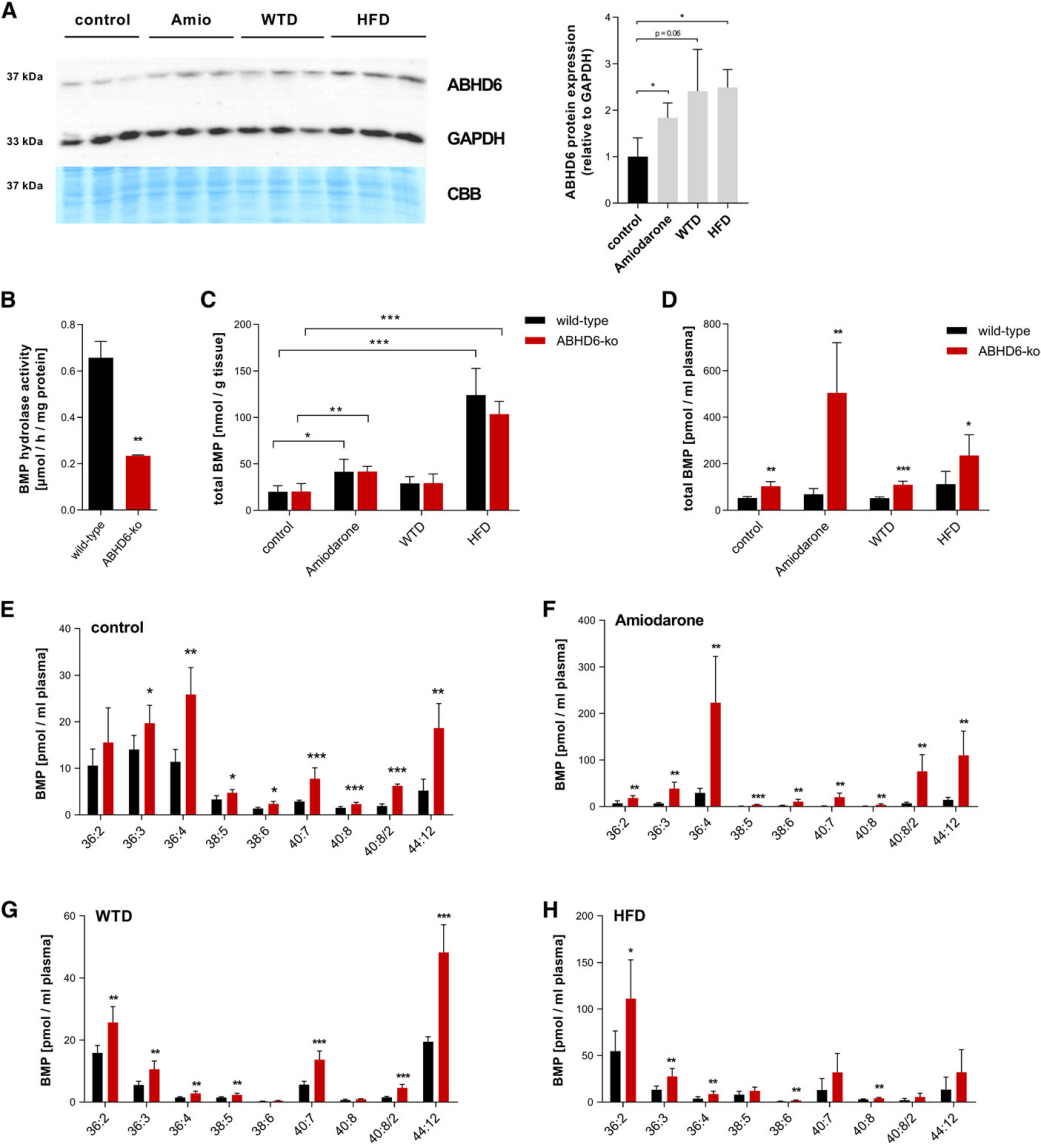
Analysis of BMP concentrations in ABHD6 KO mice. A: Immunoblot of ABHD6 of liver lysates of WT mice upon different treatments, with GAPDH and CBB as a loading control. Signal densities were assessed using ChemiDoc software (*n* = 3). B: BMP hydrolase activity in liver samples of WT and ABHD6 KO mice (*n* = 3). Total liver (C) and plasma (D) BMP content of WT and ABHD6 KO mice upon different treatments (*n* = 4–6). Plasma BMP concentrations of WT and ABHD6 KO mice fed a chow diet (control) (E), chow diet containing 100 mg/kg/day amiodarone for 3 days (F), WTD for 3 months (G), or HFD for 3 months (H). Data are presented as means + SDs (*n* = 5–6). Statistical significance was evaluated by unpaired two-tailed Student’s *t*-test. *P < 0.05, **P < 0.01, and ***P < 0.001. CBB, Coomassie Brilliant Blue.

Thomas et al. (17) reported that HFD feeding in mice leads to the increased expression of ABHD6 in the liver and other tissues. In accordance with these data, we found that the HFD as well as the WTD and amiodarone led to increased protein expression of ABHD6 in the liver compared with chow-fed controls (**Fig. 3A**). The loss of ABHD6 activity resulted in a 70% decrease in BMP hydrolase activity detected in liver lysates (Fig. 3B). These observations indicate that ABHD6 substantially contributes to hepatic BMP degradation under basal and pathophysiological conditions. However, hepatic BMP content in ABHD6 KO mice remained unchanged with the chow diet and different treatments compared with the respective WT controls (Fig. 3C). Importantly, however, we found that ABHD6 deficiency is associated with increased circulating BMP concentrations in mice that were fed the chow diet (2-fold), the chow diet supplemented with amiodarone (6-fold), and the WTD (2-fold) and HFD (2-fold) (Fig. 3D). In ABHD6 KO mice that were fed the chow diet, most of the detected BMP species were increased (Fig. 3E). Amiodarone-treated ABHD6 KO mice showed a strong increase in BMP 36:4, 40:8/2, and 44:12 (Fig. 3F). ABHD6 KO mice fed the WTD or HFD showed an increase in several BMP subspecies. Most prominent were 44:12 and 36:2, respectively (Fig. 3G, H). Elevated plasma BMP concentrations, as observed in ABHD6 KO mice, can indicate an LSD (8). Thus, we investigated whether ABHD6 KO mice exhibit changes in lysosome morphology. An analysis of liver samples by electron microscopy (supplemental Fig. S3A**)** and laser-scanning microscopy using a specific label for acidic organelles (supplemental Fig. S3B) revealed no differences between genotypes. This suggests that genetic ABHD6 deficiency promotes BMP release into the circulation but is not associated with hepatic BMP accumulation and with the formation of the multilamellar bodies characteristic of LSDs (10).

### Plasma BMP associates with HDL

**Fig. 4.**
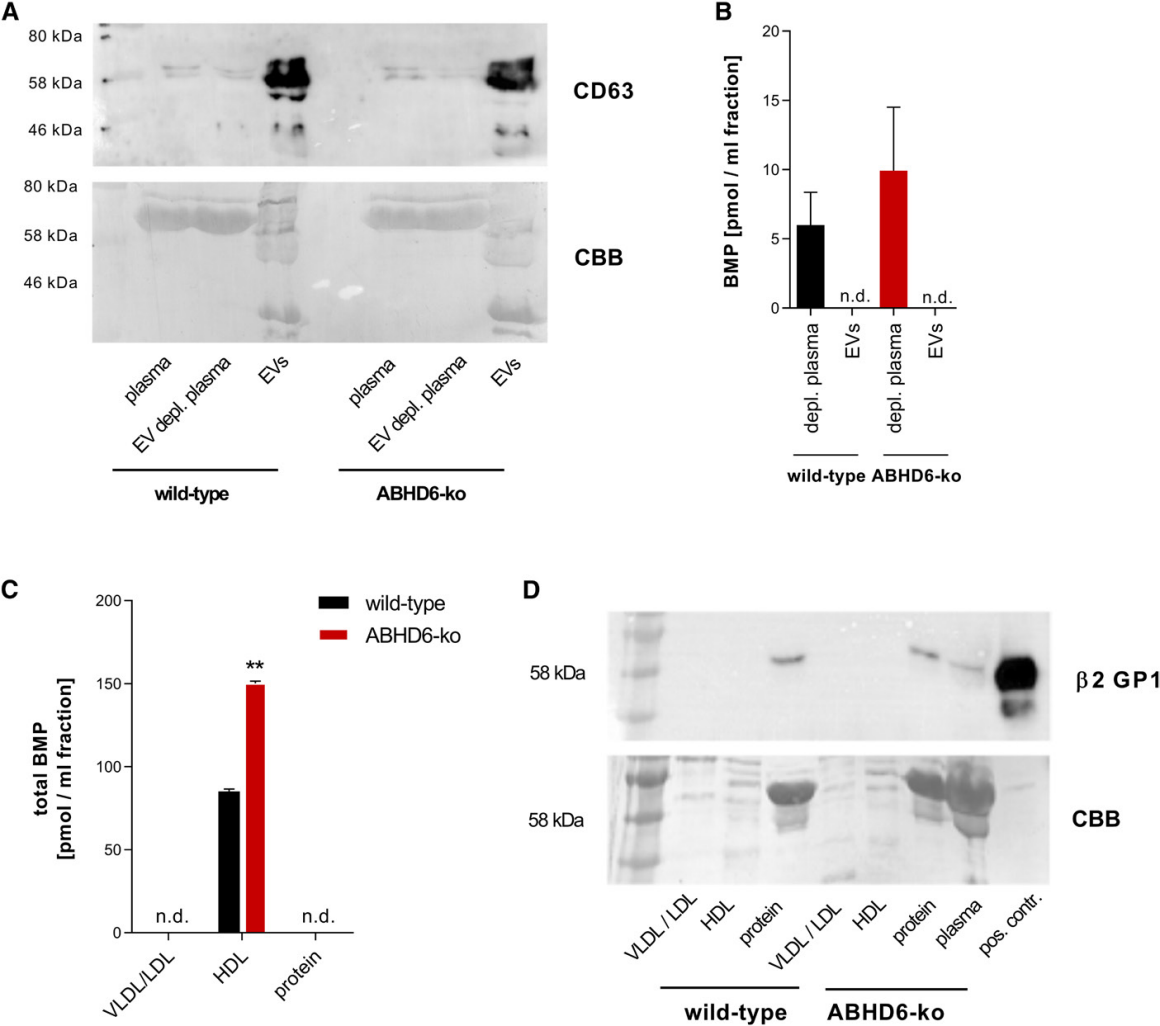
Analysis of BMP content in plasma fractions. A: Immunoblot of CD63 of plasma, exosome-depleted plasma, and exosome-enriched preparations (EVs) of WT and ABHD6 KO mice. B: BMP content in exosome-depleted plasma and exosome-enriched preparations (EVs) of WT and ABHD6 KO mice (*n* = 3). C: BMP concentrations in plasma fractionation obtained by FPLC gel filtration. D: Immunoblot of β2GPI in plasma fractions with CBB staining as a loading control. Data are presented as means + SDs. Statistical significance was evaluated by unpaired two-tailed Student’s *t*-test. **P < 0.01. CBB, Coomassie Brilliant Blue; EV, extracellular vesicle.

The mechanism of BMP release into the circulation is poorly understood. It was recently demonstrated that BMP is secreted from neurons via exosomes (26). We thus isolated an exosome-enriched fraction from the plasma of WT and ABHD6 KO mice by ultracentrifugation (27) and confirmed their enrichment using CD63 immunoblotting (**Fig. 4A**). However, BMP remained in the exosome-depleted plasma fractions of both WT and ABHD6 KO mice (Fig. 4B). Further fractionation of plasma via FPLC gel filtration revealed similar lipoprotein profiles in both genotypes, and a subsequent analysis showed that BMP is present in the HDL fraction in both WT and ABHD6 KO mice (Fig. 4C). β2GPI, a highly abundant serum protein that binds acidic phospholipids, including BMP (28, 29), was not associated with the BMP-containing HDL fraction (Fig. 4D). Together, our observations suggest that ABHD6 deficiency is associated with increased plasma BMP concentrations. Independent of the genotype, BMP associates with HDL but not with exosomes or β2GPI.

### Altered serum BMP concentration and profile in patients with liver disease

**Fig. 5.**
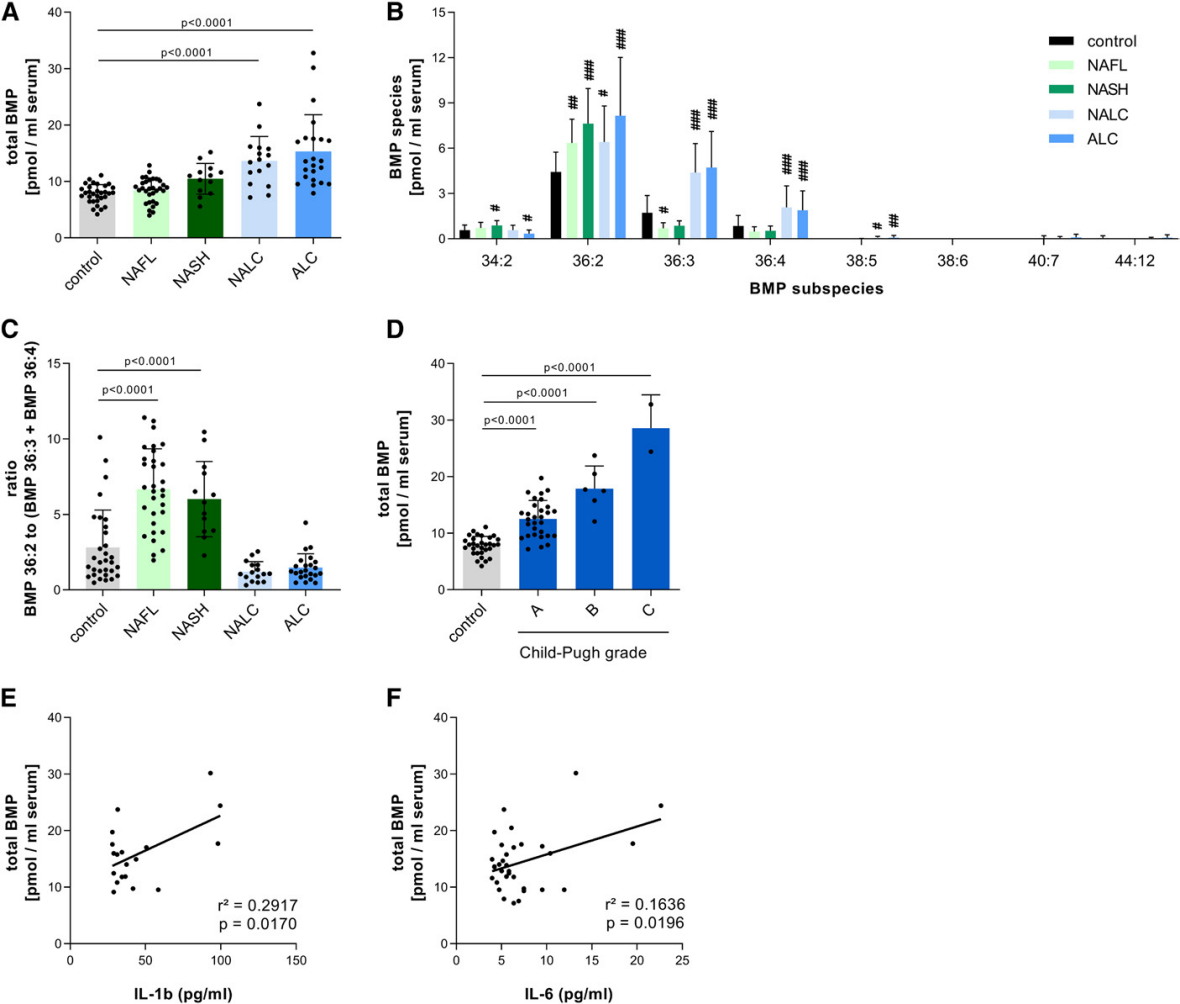
Serum BMP profile of patients with liver disease. Total serum BMP levels of healthy subjects (*n* = 32) and patients with NAFL (*n* = 31), NASH (*n* = 13), compensated NALC (*n* = 16), and compensated ALC (*n* = 23) (A) and respective BMP subspecies (B). C: Ratio of BMP 36:2 and BMP 36:3 plus BMP 36:4 in patient groups. D: Total BMP concentration in the sera of patients with NALC and ALC classified into Child-Pugh classes A–C. Correlation of total BMP levels with IL-1β (E) and IL-6 (F). Cytokines in the sera of cirrhotic patients were quantified by flow cytometry using a multiplex bead-based immunoassay. Data are presented as means + SDs. Statistical significance was evaluated by ANOVA followed by Dunnett’s post hoc test. #*P* < 0.05, ##*P* < 0.01, and ###*P* < 0.001 versus control.

Our observations in mice indicate that circulating BMP could represent a serum marker for common lipid disorders frequently associated with obesity. We thus analyzed samples from patients with liver disease, including NAFL and NASH. We also analyzed samples of patients with compensated alcoholic liver cirrhosis (ALC) and nonalcoholic liver cirrhosis (NALC). The clinical characteristics of study subjects are given in **Table 1**. Total serum BMP concentrations were unchanged in NAFL and NASH and increased in ALC and NALC (**Fig. 5A**). The major BMP species detected in human samples were 36:2, 36:3, and 36:4 (Fig. 5B). BMP 36:2 moderately increased in NASH and ALC compared with healthy controls. BMP 36:3 and 36:4 showed a modest decrease in NAFL and NASH and a robust increase in NALC and ALC. Accordingly, the ratio of BMP species, 36:2/(36:3 + 36:4), was significantly higher in NAFL and NASH compared with controls and patients with cirrhosis (Fig. 5C). In cirrhotic patients, total serum BMP levels correlated with disease severity indicated by the Child-Pugh grade (Fig. 5D) and serum IL-1β (Fig. 5E) and IL-6 (Fig. 5F) levels. These observations indicate that liver disease of a different degree and origin is associated with distinct changes in serum BMP concentration and/or BMP composition.

**TABLE 1.** Clinical characteristics of patients

	Control	NAFL	NASH	NALC	ALC
*n*	31	31	13	16	23
Age, years	53 (41–62)	47 (31–58)	56 (46–62)	56 (52–66)	58 (53–64)
Males/females, *n*	19/12	21/10	7/6	7/9	17/6
Albumin, g/dl	—	4.7 (4.4–4.8)	4.5 (4.3–4.8)	4.4 (3.8–4.8)	4.0 (3.8–4.4)
Bilirubin, mg/dl	—	0.8 (0.5–0.9)	0.7 (0.5–1.0)	1.1 (0.5–2.2)	1.3 (0.8–2.4)
Creatinine, mg/dl	—	0.9 (0.8–1.0)	0.8 (0.7–1.0)	0.8 (0.6–0.9)	0.8 (0.7–1.0)
Alanine aminotransferase, U/l	—	57 (31–90)	121 (44–185)	28 (19–59)	35 (22–40)

Values are given as medians with the interquartile range in parentheses.

### Human ABHD6 deficiency is associated with increased circulating DHA-containing BMP subspecies

**Fig. 6.**
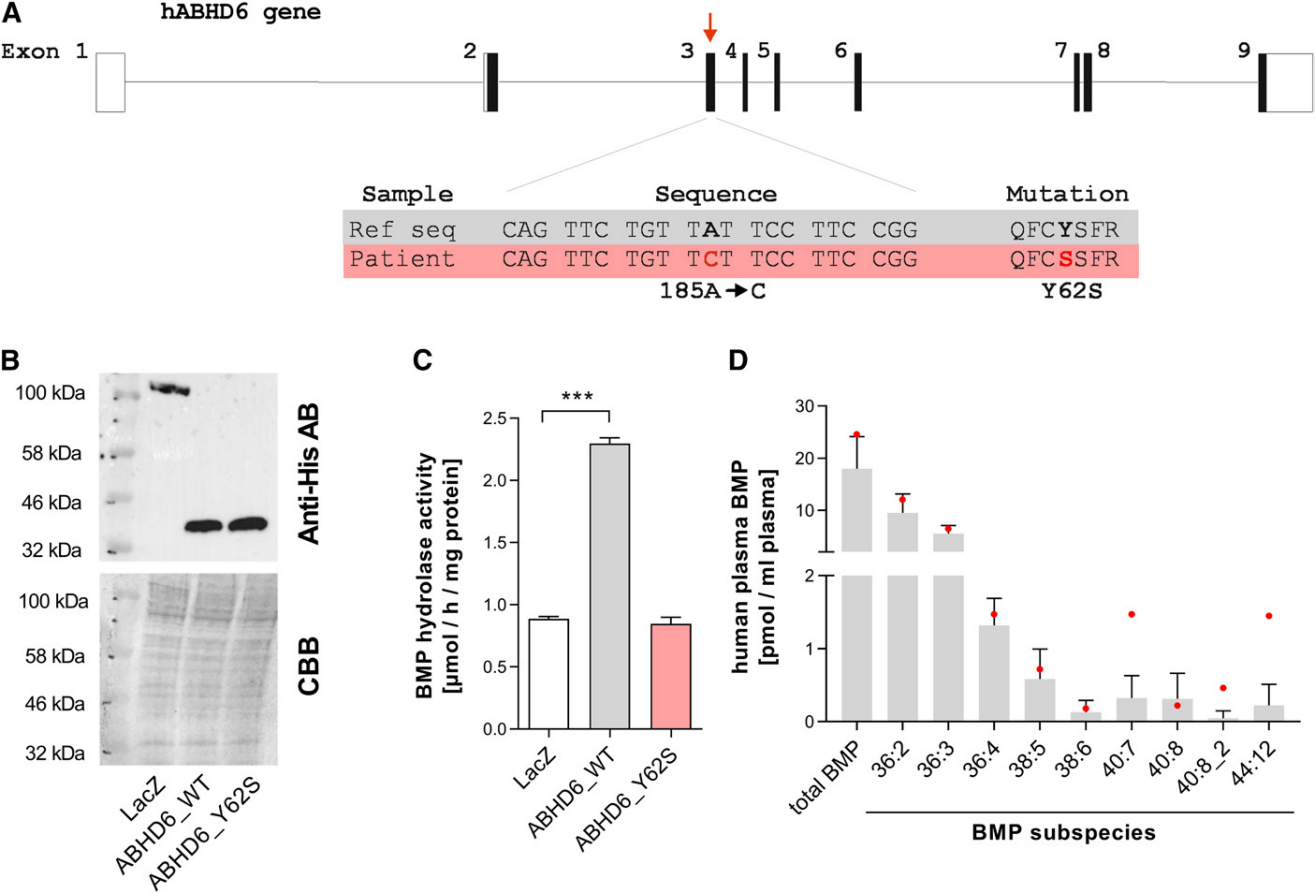
Serum BMP profile of a patient with ABHD6 deficiency. A: A single-nucleotide exchange from adenosine to cytidine at position 185 in the coding sequence of the *ABHD6* gene of a patient leads to an amino acid exchange from tyrosine (Y) to serine (S) at position 62. Immunoblot (B) and BMP hydrolase activity (C) of lysates from COS7 cells overexpressing LacZ, WT ABHD6, or mutated ABHD6 Y62S. Statistical significance was evaluated by unpaired two-tailed Student’s *t*-test. ***P < 0.001. D: Total BMP content and BMP subspecies in the serum of healthy control patients (*n* = 11) from the same population as the ABHD6-deficient patient (*n* = 1; red dots). AB, antibody; CBB, Coomassie Brilliant Blue.

A recent genetic screen in consanguineous populations (30) identified a patient with a homozygous single-nucleotide substitution in the *ABHD6* gene, leading to an amino acid substitution (Y62S) in the ABHD6 protein (**Fig. 6A**). Because it remained unclear whether this is a loss-of-function mutation, we cloned this ABHD6 variant and expressed WT and mutated ABHD6 in COS7 cells (Fig. 6B). The expression of WT ABHD6 led to a 2.5-fold increase in BMP hydrolase activity compared with control cells expressing LacZ. In contrast, the overexpression of Y62S did not increase BMP hydrolase activity, demonstrating a complete loss of enzyme activity (Fig. 6C). Additionally, we analyzed the circulating BMP profile of this patient compared with healthy controls from the same population. The predominant BMP species in human serum (18:2/3/4) remained unchanged. However, we observed a 4-, 10-, and 6-fold increase in DHA-containing BMP subspecies 40:7, 40:8/2, and 44:12, respectively, in the patient’s serum (Fig. 6D). These observations indicate that ABHD6 affects the circulating BMP profile in humans.

## DISCUSSION

Lysosomes are the main degradative cellular organelles essential for the maintenance of metabolic homeostasis and health. BMP is a major component of ILVs of late endosomes/lysosomes facilitating the degradation and sorting of cargo lipids (5). It contains high amounts of DHA and other PUFAs, indicating that it may play a role in PUFA sorting in acidic organelles. BMP accumulates in genetic and drug-induced LSDs in various tissues and is also elevated in the circulation, indicating that lysosomal stress of a different origin affects BMP metabolism. Despite its central role in lipid sorting and its association with human disease, very little is known about BMP metabolism in common lipid-associated disorders other than LSDs, such as obesity, nonalcoholic fatty liver disease (NAFLD), and atherosclerosis. Published work suggests that dietary lipid overload increases the hepatic (13, 31) and renal BMP content in mice (32) and that serum BMP concentrations in humans are increased in ALC (33).

HFDs and WTDs are frequently used experimental diets for inducing metabolic disorders in mice. Here we show that these diets substantially affect BMP concentrations in the liver and plasma of mice. The HFD had even more pronounced effects on BMP metabolism than amiodarone, which is considered as a prototypical drug inducing LSDs (10). Strikingly, changes in BMP concentrations and FA composition allowed a clear distinction between treatments, suggesting that the serum BMP profile could represent a useful diagnostic marker for common lipid-associated disorders such as NAFLD, which affects 25% of the global adult population. Because serum biomarkers for diagnosis and monitoring of NAFLD are urgently required (34), we analyzed the serum BMP profile of patients with different stages of liver disease. Changes in BMP profiles were less pronounced as observed in mice fed the HFD or WTD. NAFL and NASH were associated with alterations in FA composition of the major circulating BMP species (36:2/3/4). These observations indicate that liver steatosis induces moderate changes in BMP metabolism independent of disease progression. We found no differences in BMP concentrations and FA composition between NAFL and NASH, indicating that an analysis of circulating BMP is not sufficient for monitoring disease progression. In cirrhotic patients, we observed increased plasma BMP concentrations in accordance with published data for ALC (33), mainly due to an increase of the BMP subspecies 36:3/4. Notably, plasma BMP concentrations in cirrhotic patients correlated with disease severity, and BMP might thus represent an interesting marker for end-stage liver disease.

The observed changes in BMP content and FA composition require enzymes catalyzing synthesis, degradation, and/or remodeling of BMP. Currently, little is known about the anabolic and catabolic pathways of BMP metabolism (35). We previously demonstrated that the serine hydrolase ABHD6 exhibits BMP hydrolase activity and is responsible for most of the BMP hydrolase activity detected in liver lysates (13). ABHD6 is located at the ER and on the cytosolic side of outer membranes of late endosomes/lysosomes. The enzyme may be involved in remodeling outer membranes of late endosomes/lysosomes by hydrolyzing BMP and possibly other lipid substrates exported from these organelles (13). The enzyme is upregulated in the liver in response to lysosomal stress induced by amiodarone and lipid-enriched diets, suggesting that it affects tissue BMP levels. Surprisingly, however, we observed unchanged hepatic BMP concentrations in ABHD6 KO mice, while circulating BMP concentrations were elevated. The lack of ABHD6 also potentiated amiodarone-, WTD, and HFD-induced elevations in plasma BMP, indicating that nonhydrolyzed BMP is released into the circulation.

To better understand the export mechanism of BMP, we examined whether circulating BMP is associated with exosomes, lipoproteins, or plasma proteins. Despite its high enrichment in ILVs, which represent precursors of exosomes (3), we exclusively found BMP in the HDL fraction of WT and ABHD6 KO mice. This suggests that BMP is rather released via HDL/ApoA1-dependent mechanisms, such as ABC transporters or scavenger receptor class B type 1, than via the exosomal pathway. In comparison, studies in humans have suggested that approximately 40% of plasma BMP is associated with lipoproteins but only 9% are found in the HDL fraction, indicating differences in BMP metabolism between species (8). Further studies are necessary to clarify the mechanism of BMP release and its transport in the circulation.

Human and mouse ABHD6 are capable of hydrolyzing BMP in vitro, and several point mutations in the human *ABHD6* have been reported that lead to the loss of enzyme activity (13). A recent genetic study (30) in consanguineous populations identified candidate variants in 75 genes that were previously not associated with human disease. The *ABHD6* gene was among these candidate variants, with a homozygous single-nucleotide substitution in the coding region leading to an amino acid substitution (Y62S). The observed phenotype of the patient includes fine motor delay, speech delay, intellectual disability, and autistic features. It is currently unclear whether the *ABHD6* mutation is causal for the phenotype. Our data demonstrate that this *ABHD6* variant lacks BMP hydrolase activity. Furthermore, an analysis of the patient’s plasma revealed increased DHA-containing BMP subspecies. Notably, these were also the most elevated subspecies in ABHD6-deficient mice, indicating that ABHD6 affects BMP metabolism and release similarly in mice and humans.

ABHD6 was originally described as an MG hydrolase degrading the endocannabinoid 2-AG in addition to MG lipase, the major enzyme catalyzing 2-AG hydrolysis (25). Subsequent studies revealed that the inactivation of ABHD6 protects against HFD-induced obesity, liver steatosis, and insulin resistance (17, 18). In accordance with these data, our results showed reduced body weight and hepatic steatosis in ABHD6 KO mice fed the HFD. The molecular mechanism behind these protective effects is incompletely understood. Zhao et al. (18) proposed that ABHD6 deficiency promotes adipose tissue browning via the activation of PPARα/γ by *sn*-1-monoacylglycerols. Alternatively, ABHD6 could regulate inflammatory pathways by degrading prostaglandin D_2_-glycerol ester (36) or mobilizing arachidonic acid from 2-AG (37). We could not detect differences in 2-AG and other MG species in the brain, liver, and plasma of ABHD6 KO mice, indicating that the enzyme does not substantially affect tissue and circulating MG levels. Nevertheless, ABHD6-dependent degradation of MG might be important in specialized cells or cellular compartments such as pancreatic β-cells (15) or postsynaptic membranes of neurons (14). Another study identified ABHD6 as a component of α-amino-3-hydroxy-5-methyl-4-isoxazolepropionic acid receptor (AMPAR) complexes (16). These complexes regulate the trafficking and functions of AMPARs that are localized in postsynaptic terminals, where they mediate fast excitatory neurotransmission and synaptic plasticity. Changes in endocannabinoid or AMPAR signaling may affect neurotransmission, resulting in behavioral defects. Accordingly, we performed several standard behavioral tests but could not find any changes in behavioral, learning, and memory performance.

In summary, we demonstrate that commonly used atherogenic and obesity-inducing diets cause robust changes in hepatic and circulating BMP concentrations in mice. Considering that BMP is crucial for lysosomal lipid sorting and accumulates in LSDs, diet-induced alterations in BMP likely reflect lysosomal stress triggered by an increased lipid flux. In humans, liver disease alters the serum BMP profile depending on disease progression. Furthermore, we demonstrate that the BMP hydrolase ABHD6 affects circulating BMP profiles in mice and humans. Further studies are needed to elucidate the molecular pathways mediating BMP synthesis, degradation, and release. Understanding these processes will provide new insights into the regulation of lysosomal function as well as the pathogenesis of lysosomal dysfunction.

## Supplementary Material

Supplemental Data
