# Supplementary material for: Metabolic disease and ABHD6 alter the circulating bis(monoacylglycerol)phosphate profile in mice and humans
Source: J Lipid Res. 2019 Mar 20;60(5):1020–31. doi: 10.1194/jlr.M093351 (PMC6495172; doi:10.1194/jlr.M093351)
Supplement: Supplemental Data [file supp_60_5_1020__index.html]

Metabolic disease and ABHD6 alter the circulating bis(monoacylglycerol)phosphate profile in mice and humans — Metabolic disease and ABHD6 alter the circulating bis(monoacylglycerol)phosphate profile in mice and humans — Supplemental Data 

# Metabolic disease and ABHD6 alter the circulating bis(monoacylglycerol)phosphate profile in mice and humans

## Supplemental Data

- Supplemental information (.pdf, 331 KB) - Metabolic and behavioural characterization of ABHD6-ko mice. Lysosomal morphology in ABHD6-ko mice.
