# Supplementary material for: Metabolic disease and ABHD6 alter the circulating bis(monoacylglycerol)phosphate profile in mice and humans
Source: J Lipid Res. 2019 Mar 20;60(5):1020–31. doi: 10.1194/jlr.M093351 (PMC6495172; doi:10.1194/jlr.M093351)
Supplement: Supplemental Data [file 10.1194_M093351_jlr.M093351-1.pdf]

SUPPLEMENTAL INFORMATION:

Metabolic disease and ABHD6 alter the circulating bis(monoacylglycerol)phosphate profile in mice and humans

Gernot F. Grabner[1,10], Nermeen Fawzy[1,9,10], Maria A. Pribasnig[1], Markus Trieb[2], Ulrike Taschler[1], Michael Holzer[2], Martina Schweiger[1], Heimo Wolinski[1], Dagmar Kolb[3], Angela Horvath[4], Rolf Breinbauer[5,9], Thomas Rülicke[6], Roland Rabl[7], Achim Lass[1,9], Vanessa Stadlbauer[4], Birgit Hutter-Paier[7], Rudolf E. Stauber[4], Peter Fickert[4], Rudolf Zechner[1,9], Gunther Marsche[2,9], Thomas O. Eichmann[1,8,9,*], and Robert Zimmermann[1,9,11*]

[1] Institute of Molecular Biosciences, University of Graz, Graz, Austria

[2] Division of Pharmacology, Otto Loewi Research Center, Medical University of Graz, Graz, Austria

[3] Core Facility Ultrastructure Analysis, Division of Cell Biology, Histology and Embryology, Gottfried Schatz Research Center, Medical University of Graz, Graz, Austria

[4] Division of Gastroenterology and Hepatology, Department of Internal Medicine, Medical University of Graz, Graz, Austria

[5] Institute of Organic Chemistry, Graz University of Technology, Graz, Austria

[6] Institute of Laboratory Animal Science, University of Veterinary Medicine Vienna, Vienna, Austria

[7] Center for Explorative Lipidomics, BioTechMed-Graz, Graz, Austria

[8] BioTechMed-Graz, Graz, Austria

SUPPLEMENTAL FIGURES:

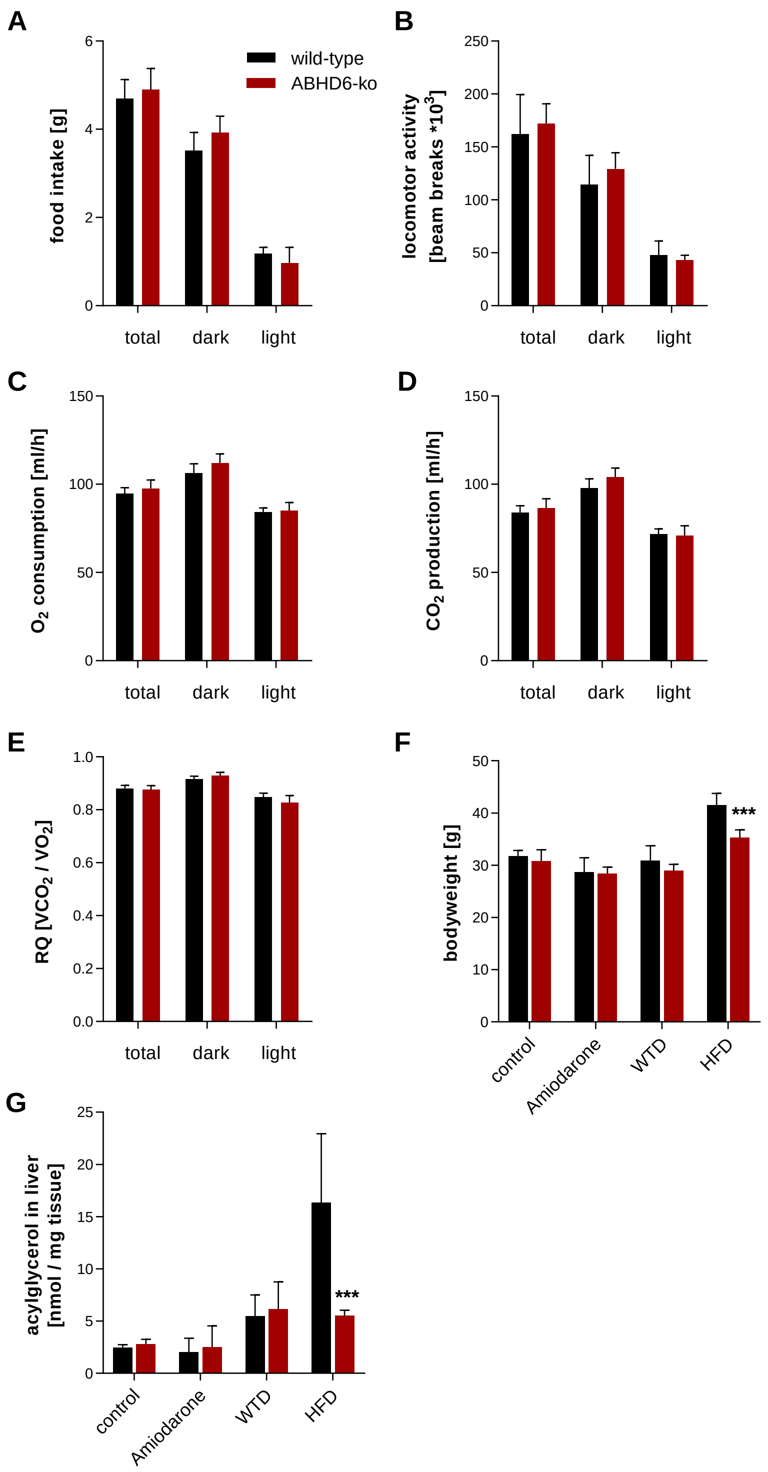

Supplemental Figure S1: Gross metabolic characterization of ABHD6-ko mice. (A) Food consumption, **(B)** locomotor activity, **(C)** O_2 consumption, **(D)** CO_2 production, and **(E)** respiratory quotient (RQ) of wild-type and ABHD6-ko mice were calculated from a 96 h measurement period in a

laboratory animal monitoring system (LabMaster, TSE Systems, Germany). **(F)** Bodyweight and **(G)** liver acylglycerol content of mice fed chow diet (control), 100 mg/kg/day Amiodarone for 3 days, western-type diet (WTD) for 3 months, or high-fat diet (HFD) for 3 months. Data are presented as means + S.D. (n=5-7). Statistical significance was evaluated by unpaired two-tailed Student's *t* test. ***$p < 0.001$.

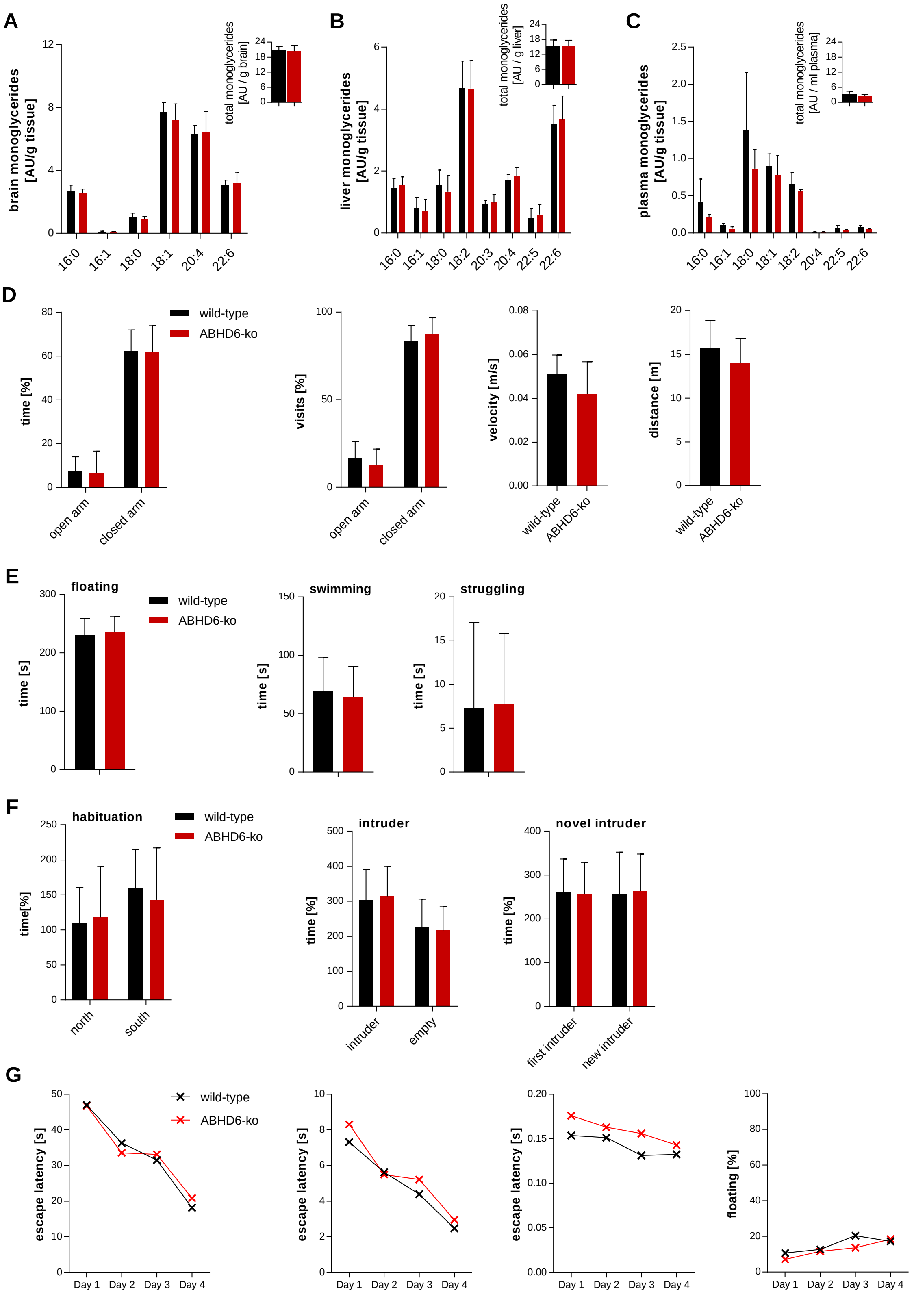

Supplemental Figure S2: Behavioral characterization of ABHD6-ko mice. Monoglycerides were measured from **(A)** brain, **(B)** liver, and **(C)** plasma of wild-type and ABHD6-ko mice via UPLC-MS (n=4–6). Mice were tested for **(D)** anxiety-like behavior in the elevated plus maze and **(E)** depression-

like behavior in forced swim test. **(F)** Social memory was analyzed via Three-Chamber-Social test and **(G)** spatial memory via Morris water maze. Data are presented as means + S.D. (n=10).

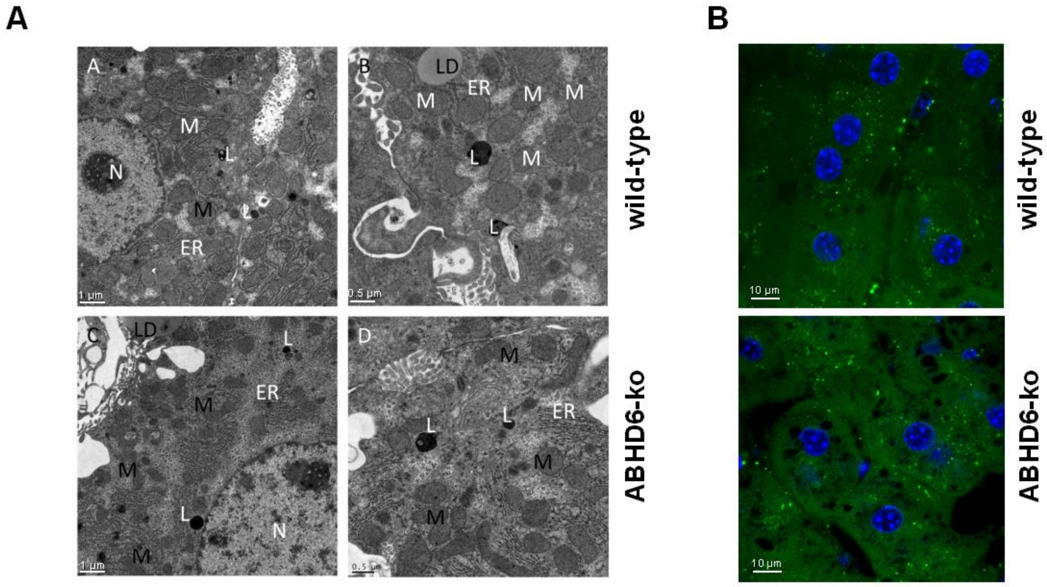

Supplemental Figure S3: Lysosomal morphology in ABHD6-ko mice. Lysosome morphology of wild-type and ABHD6-ko was analyzed via **(A)** electron microscopy or **(B)** confocal laser scanning microscopy of liver samples stained for lysosomes with lysotracker (green) and for nuclei with DAPI (blue).

SUPPLEMENTAL METHODS:

MG analysis

Total lipids of weighed tissue explants were extracted twice as described for BMP analysis using 5.8 nmol 17:0 MG as internal standards (Avanti Polar Lipids) per sample. The combined lipid-containing organic phase was dried under a stream of nitrogen and MGs were isolated by solid phase extraction using an amino-functionalized silica gel column (750 µg bulk material/column, Phenomenex). Lipids were loaded in a total of 1 ml chloroform and fractions were obtained by consecutive elution with hexane/ethyl acetate (85/15, v/v, 4x1 ml, F1), chloroform/methanol (23/1, v/v, 4x1 ml, F2), and chloroform/methanol/sodium acetate (30/60/8, v/v/v, 4x1 ml, F3). Combined F2 and F3 including MGs were evaporated and resolved in 50 µl 2-propanol/chloroform/methanol (70/20/10, v/v/v) for LC-MS analysis. Chromatographic separation of MG species was modified after Knittelfelder et al. using an AQUITY-UPLC system (Waters Corporation) equipped with a HSS T3 column (2.1x50 mm, 1.8 µm; Waters Corporation) (23) with solvents as described for BMP analysis. A Synapt G1-qTOF mass spectrometer (Waters Corporation) equipped with an ESI source was used for detection. Data acquisition was done by MassLynx 4.1 software (Waters Corporation) and MGs were analyzed using the “Lipid Data Analyzer 1.6.2” software (24). Data were normalized for recovery, extraction-, and ionization efficacy by calculating analyte/ISTD ratios (AU) and expressed as AU/g tissue.

Behavioral evaluation

At an age of 3 to 5 months, male ABHD6-ko and wild type littermates (n=10 per group) were tested in behavioral setups as the Elevated Plus Maze (EPM), the Three-Chamber-Social Test (3CS), the Morris Water Maze (MWM) and the Forced Swim Test (FST), to assess effects of ABHD6 gene knock-out on emotional, social and cognitive function.

<u>Elevated Plus Maze</u>: To evaluate anxiety levels the EPM was performed. The EPM was set at a height of 50 cm and consisted of four arms, each 5 cm wide and 30 cm long with two opposing open arms and two opposing closed arms (sheltered arms, with walls, 15 cm high). The test took place under red light illumination. On the test day, animals were brought into the experimental room one hour prior to

testing. The mouse was placed in the center area facing the open arm. The behavior during the test session was recorded for five minutes and behavioral parameters such as time spent in the open and in the closed arms, number of visits in the open and closed arms as well as latency to enter the open arm were calculated. Data were generated using Noldus Ethovision XT.

Three-Chamber-Social Test: This test was performed to monitor a possible lack of sociability. In detail, the test evaluates the preference for spending time with an unfamiliar mouse compared to an empty novel object and evaluates the preference for an unfamiliar versus a familiar mouse. Two stranger mice, that had never been in physical contact with the test mouse before, were used per arena for the entire experimental phase.

The Three Chamber Social test apparatus consists of a box separated into three chambers, a middle chamber (170x260x290mm) and two side chambers with the same size (280x260x290mm). The chambers were separated by two clear plastic dividers and connected by open doorways. The quadratic wire cages, which contained the social interaction partners were 90x90mm large and were located in the side chambers. After each testing the apparatus was cleaned with paper towels and 70% ethanol. Subject mice were habituated in the test room for one hour prior to the start of the experiment. The test was performed under red light illumination.

Social approach test: Test mice were habituated in the empty apparatus for five minutes ultimately before the test phase started. The five minutes habituation phase prior to the social approach test was videotaped to monitor a possible preference for one direction. After this phase an unfamiliar adult mouse of the same sex and approximately same age as the test animal was placed inside the small cage in one chamber. In the other partition an identical empty wire cage was placed. The test mouse was allowed to move freely between the three chambers for ten minutes.

Social novelty test: The social recognition test was performed after the social approach test. In a ten minutes phase the preference to social novelty was quantified. The no longer unfamiliar mouse remained in its wire cage and an unfamiliar mouse was placed in the empty cage. Now the test mouse was allowed to move freely between the three chambers for ten minutes.

The whole test was videotaped. The time the test mouse spent in each chamber was evaluated during habituation, social approach and social recognition. Data were generated by using Noldus Ethovision XT.

Morris Water Maze: Spatial learning and memory performance was assessed in the MWM. The MWM was performed using the following pattern: Four trials on each of four consecutive days. In all trials, the platform was located in the northeast (NE) quadrant of the pool and mice started from predefined positions (southeast (SE), southwest (SW), northwest (NW)) in a pseudorandom manner. A single trial lasted for a maximum of 60 seconds. In case the mouse did not find the hidden, diaphanous platform within this time, the experimenter guided the mouse to the target. Mice were allowed to rest on the platform for 10-15 sec to orientate in the surrounding. 24 hours after the last trial on day 4, mice were tested in the probe trial (PT) for 60 seconds. During the PT, the platform was removed from the pool and the number of crossings over the former target position as well as the abidance in the target quadrant was recorded. For quantification of the escape latency (the time to find the hidden platform), of pathway (the length of the trajectory to reach the target), of target zone crossings and of the abidance in the target quadrant in the PT), a computerized video tracking system (Biobserve, Viewer III) was used.

Forced Swim Test: To measure helplessness and depressive-like behavior, The FST was performed. The Porsolt Forced Swim Test (FST) is based on the assumption that animals will try to escape from an aversive stimulus.

Animals were placed in a transparent Plexiglas cylinder (20cm diameter) filled with water (about 21°C) for a 5 minute lasting test session. The behavior was videotaped and the duration of immobility as well as struggling and swimming during the whole 5 minutes session was calculated using an automated system (Noldus, Ethovision).

Fluorescence microscopy

Microscopy was performed using a Leica SP5 confocal and 2-photon microscope with spectral detection (Leica Inc., Germany) as well as a Leica HCX PL APO 63x NA 1.4 OIL immersion objective. Cells were stained using DAPI (Invitrogen, Inc.; final concentration 300nM) and LysoTracker Green (Invitrogen, Inc.; final concentration 50nM). DAPI was excited with a 2-photon laser source and emission detected using appropriate filters. MitoTracker Green was exited using an argon laser at 488 nm and emission detected between 500-550 nm. DAPI and LysoTracker images were acquired sequentially.

Electron microscopy

Mice were transcardially perfused with 4% Paraformaldehyde in PBS. Tissues were excised and fixed in 2.5% glutaraldehyde and 2% paraformaldehyde in PBS for 2 h, post-fixed in 2% osmium tetroxide for 2 h at RT, dehydrated in graded series of ethanol, embedded in TAAB (Agar Scientific, Essex, GB) epoxy resin and allowed to polymerise for 48 h at 60°C.

Ultrathin sections (70 nm) were cut with a UC 7 Ultramicrotome (Leica Microsystems, Wetzlar, Germany) and stained with lead citrate for 5 min and with uranyl acetate (UAc) for 15 min. Images were taken using a Tecnai G2 20 transmission electron microscope (FEI, Eindhoven, Netherlands) with a Gatan ultrascan 1000 charge coupled device (CCD) camera (temperature −20°C; acquisition software Digital Micrograph; Gatan, Munich, Germany). Acceleration voltage was 120 kV.
